# Commentary: Coronary artery mycotic aneurysm in a patient suffering from subacute endocarditis: a case report and literature review

**DOI:** 10.3389/fcvm.2023.1286416

**Published:** 2023-11-28

**Authors:** Parvin Kalhor, Tahere Davarpasand

**Affiliations:** Tehran Heart Center, Tehran University of Medical Sciences, Tehran, Iran

**Keywords:** coronary artery mycotic aneurysm, bicuspid aortic valve (BAV), coronary artery ectasia (CAE), infective endocarditis (IE), congenital anomalies

A Commentary on Coronary artery mycotic aneurysm in a patient suffering from subacute endocarditis: a case report and literature review By Parvin Kalhor and Tahereh Davarpasand (2023). Front. Cardiovasc. Med. 10:1286416. doi: 10.3389/fcvm.2023.1286416

We read the case report titled “Coronary artery mycotic aneurysm in a patient suffering from subacute endocarditis: a case report and literature review” by Hali et al. (1) with great interest. In their study they reported a 42-year-old man who was referred with a 2-month history of feverishness and embolic ischemic left cerebellar infarction. He was diagnosed with infective endocarditis (IE) based on the results of echocardiography and blood culture by *Viridans Streptococci* organism. One of his echocardiographic findings was a bicuspid aortic valve (BAV) without aortic dilation. Before the surgery, a contrast-enhanced computed tomography (CT) scan revealed an irregular dilation of the left main coronary artery extending from the left coronary sinus to the proximal part of the left anterior descending artery. A diagnosis of coronary artery mycotic aneurysm (CAMA) was considered for him, but not without concerns.

Septic condition and contrast-enhanced CT findings such as aneurysmal dilatation of the left main coronary artery with a diameter of 12.7 mm with an irregular border raised clinicians’ suspicion about CAMA in this patient; however, our concerns regarding this diagnosis are strong. It is more probable that the patient is suffering from both BAV and CAA, caused by congenital underlying causes that have unfortunately been followed by endocarditis at this age.

CAMA is a rare and potentially fatal diagnosis that based on what is currently known, is considered a clinical diagnosis for patients.

A study conducted by Restrepo et al. (2) on 55 CAMA patients showed that CAMA mostly occurred in men, the right coronary artery was the most affected vessel, and about 53.3% of the time, Staphylococcus aureus was the responsible organism. Some imaging findings may also be helpful to make us more fully suspect CAMA. A large lobulated or saccular shape aneurysm with mural thrombosis and thickened wall with tissue stranding around affected vessels in contrast-enhanced CT, in combination with an infective setting like fever, bacteremia, infective endocarditis, septic emboli, or recent interventional procedure, can raise our suspicion for CAMA diagnosis. However it is possible that the involvement of the left coronary system with the *Viridans Streptococci* organism, without any wall thickening or tissue stranding around the affected vessel, could suggest a different diagnosis as being more likely than CAMA.

Given the fact that the aortic valve and the proximal portion of the coronary arteries share a common embryonic origin, a study was performed to assess the coincidence of these two congenital anomalies by Meindl et al. (3). The study found that patients with BAV are twice as likely to have coronary artery ectasia (CAE), regardless of aortic dilation.

It is crucial to be aware of the potential complications of CAMA, including coronary artery rupture, cardiac tamponade, fistula formation, distal embolization-induced ischemia/infarction, and sudden cardiac death, with a consecutive mortality rate of about 43%–53% according to the rarity literature's review (4).

We are concerned about the accuracy of the patient’s CAMA diagnosis due to their benign progression during the first year of follow-up.
